# Comparison of circulating lipid profiles between fasting humans and three animal species used in preclinical studies: mice, rats and rabbits

**DOI:** 10.1186/s12944-015-0104-4

**Published:** 2015-09-10

**Authors:** Masaki Ishikawa, Kosuke Saito, Masayo Urata, Yuji Kumagai, Keiko Maekawa, Yoshiro Saito

**Affiliations:** Division of Medicinal Safety Science, National Institute of Health Sciences, 1-18-1 Kamiyoga, Setagaya, Tokyo 158-8501 Japan; Clinical Research Center, Kitasato University School of Medicine, 1-15-1 Kitasato, Minami, Sagamihara, Kanagawa 252-0374 Japan

**Keywords:** Preclinical studies, LC-MS, Circulating lipid metabolites, Lipidomic analysis

## Abstract

**Background:**

Circulating lipid metabolites are associated with many physiological and biological processes in the body, and therefore could be used as biomarkers for evaluating drug efficacy and safety in preclinical studies. However, differences in circulating lipid profiles among humans and animals often used in preclinical studies have not been fully investigated.

**Methods:**

We performed lipidomic analysis to obtain circulating lipid profiles of fasted humans (Caucasian, *n* = 15) and three animal species used in preclinical studies (mice [BALB/c, *n* = 5], rats [Sprague–Dawley, *n* = 5], and rabbits [New Zealand White, *n* = 5]) by using liquid chromatography-mass spectrometry.

**Results:**

Our data showed marked differences in lipid profiles among humans and these animal species. Furthermore, we observed that the levels of many lipid metabolites, such as poly-unsaturated fatty acid-containing cholesteryl esters, ether-type phosphoglycerolipids, and sulfatides, were significantly different (*p* < 0.05) by more than 10-fold in these animals (depending on the animal species) from humans.

**Conclusion:**

Our data could be useful while extrapolating the data on the biomarker candidates identified in preclinical studies into clinical studies.

**Electronic supplementary material:**

The online version of this article (doi:10.1186/s12944-015-0104-4) contains supplementary material, which is available to authorized users.

## Background

Lipid metabolites, such as phosphoglycerolipids (PLs) and sphingolipids (SLs), are components of the cell membrane, and play key roles in a variety of physiological and biological processes including apoptosis [1] and autophagy [2]. These lipid metabolites are also the major components of plasma lipoproteins, including low- and high-density lipoproteins. Because somatic tissues uptake and/or excrete lipid metabolites through lipoproteins, the levels of circulating lipid metabolites are closely related to the levels of lipid metabolites in tissues [3]. Thus, circulating lipid metabolites might be associated with many physiological and biological processes in the whole body, and therefore might help in selecting useful biomarkers for evaluating diseased states and drug responses.

Lipidomics is a metabolomics approach focused on lipid metabolites [4, 5] that can overview the lipid profile of biofluids and tissues, and hence is used as a high-throughput tool for simultaneously identifying various biomarker candidates. So far, lipidomics has already been applied not only in clinical studies [6–8], but also in preclinical pharmacology and toxicology studies [9–13]. This is because circulating lipid metabolites are common among animals used in preclinical studies and humans, and thus can be used to simplify the extrapolation of data on biomarkers identified in preclinical studies into clinical studies. However, differences in circulating lipid profiles among humans and animals used in preclinical studies have not been fully investigated.

In the present study, we employed lipidomic analysis to obtain circulating lipid profiles of humans (Caucasian, *n* = 15) and three animal species used in preclinical studies (mice [BALB/c, *n* = 5], rats [Sprague–Dawley, *n* = 5], and rabbits [New Zealand White, *n* = 5]) by using liquid chromatography-mass spectrometry (LC-MS). To minimize gender and age-associated variation affecting the differences in lipid metabolites, we selected young adult males which are generally used in the preclinical and phase I studies. We observed marked differences in lipid profiles between humans and all the selected animal species. In addition, our data showed that the levels of many lipid metabolites, such as poly-unsaturated fatty acid (PUFA)-containing cholesteryl esters (ChEs), ether-type PLs and sulfatides (Suls), are more than 10-fold higher in the selected animals (depending on the animal species) than in humans. These findings would provide useful fundamental information for selecting biomarker candidates obtained/identified in preclinical studies for future extrapolation into clinical studies.

## Material and methods

### Human plasma

Blood samples from healthy white males were purchased from PromedDX (Norton, MA). The ethics committee of the National Institute of Health Sciences authorized PromedDX as a validated provider of blood samples and exempted us from the committee’s approval for use of the purchased blood samples. The samples were collected after obtaining written informed consent from all human subjects; the subjects were 15 young men (25-33-years-old) who had been fasting for more than 14 h. Fresh blood from each individual was collected and simultaneously drawn into 10 ml Vacutainer Plasma Separator Tubes containing EDTA (Becton Dickinson, Franklin Lakes, NJ), which is needed for plasma separation. Plasma was separated within 2 h after the collection of blood samples. The plasma samples were immediately frozen and stored at −80 °C. After shipment with dry ice from PromedDX, all frozen samples were thawed once on ice and divided into small aliquots before storing at −80 °C until lipid extraction.

### Animal plasma

Plasma samples of male BALB/c mice (*n* = 5, 10-weeks-old) and male New Zealand white rabbits (*n* = 5, 10-weeks-old) were purchased from KOHJIN BIO (Saitama, Japan). The plasma sample of male Sprague–Dawley rats (*n* = 5, 8-weeks-old; Charles River Japan, Kanagawa, Japan) was prepared in our laboratory. All plasma samples were separated within 2 h after collection of blood samples by using EDTA as an anticoagulant. The plasma samples were obtained from all animals after 16 h of fasting. The plasma samples were immediately frozen and stored at −80 °C. The experimental protocols for the animal procedures were approved by the Ethics Review Committee for Animal Experimentation of the National Institute of Health Sciences (Tokyo, Japan).

### Lipid extraction

Lipid extraction and measurement of lipid metabolites by LC-MS was performed as reported previously [14, 15]. In brief, lipid metabolites were extracted from 90 μL of plasma for humans and animal species, by using the Bligh and Dyer method with a few modifications. Briefly, plasma was mixed with 3.71 mL of chloroform/methanol/20 mM Kpi (1:2:0.8) for 5 min. 12:0–12:0 phosphatidylethanolamine (12:0–12:0 PE, Avanti, Polar Lipids, Inc., Alabaster, AL) was used as the internal standard (IS). Next, 1 mL each of chloroform and 20 mM Kpi was added, mixed for 5 min, and centrifuged at 1000 × g for 10 min. After discarding upper layers, 3.2 mL of chloroform/methanol/100 mM KCl (3:47:48) were added, mixed, and then centrifuged at 1000 × g for 10 min to separate the lower organic layer. The lower organic layers were supplied to non-targeted lipidomic analysis.

### Non-targeted lipidomics

The lower organic layers were used for the measurement of PLs, SLs, and neutral lipids (NLs) using LC-time of flight (TOF)-MS (Acquity UPLC system-LCT Premier XE; Waters, Milford, MA). The samples from each group were randomized across the LC-TOF-MS measurement. PLs and SLs were detected in the negative ion mode, while NLs were detected in the positive ion mode. Data obtained from LC-TOF-MS were processed using the 2DICAL software (Mitsui Knowledge Industry, Tokyo, Japan). The extracted ion peaks were analyzed for identifying lipid metabolites by comparison of the ion features including RT, m/z, preferred adducts, and in-source fragments of the experimental samples with those of our reference library of lipid metabolite entries. The levels of the identified lipid metabolites were normalized to those of IS. If more than two lipid species possessed the same chemical formula, alphabetical letters were put after each lipid molecule class to distinguish them from each other.

### Statistical analysis

The statistical analyses were performed using Welch’s *t*-test with Bonferroni correction for the comparison of metabolite levels among humans and different animal species. Differences with *p* values less than 0.05 were considered statistically significant.

## Results and discussion

### Lipidomic analysis of human and preclinical animal plasma

**Fig. 1:**
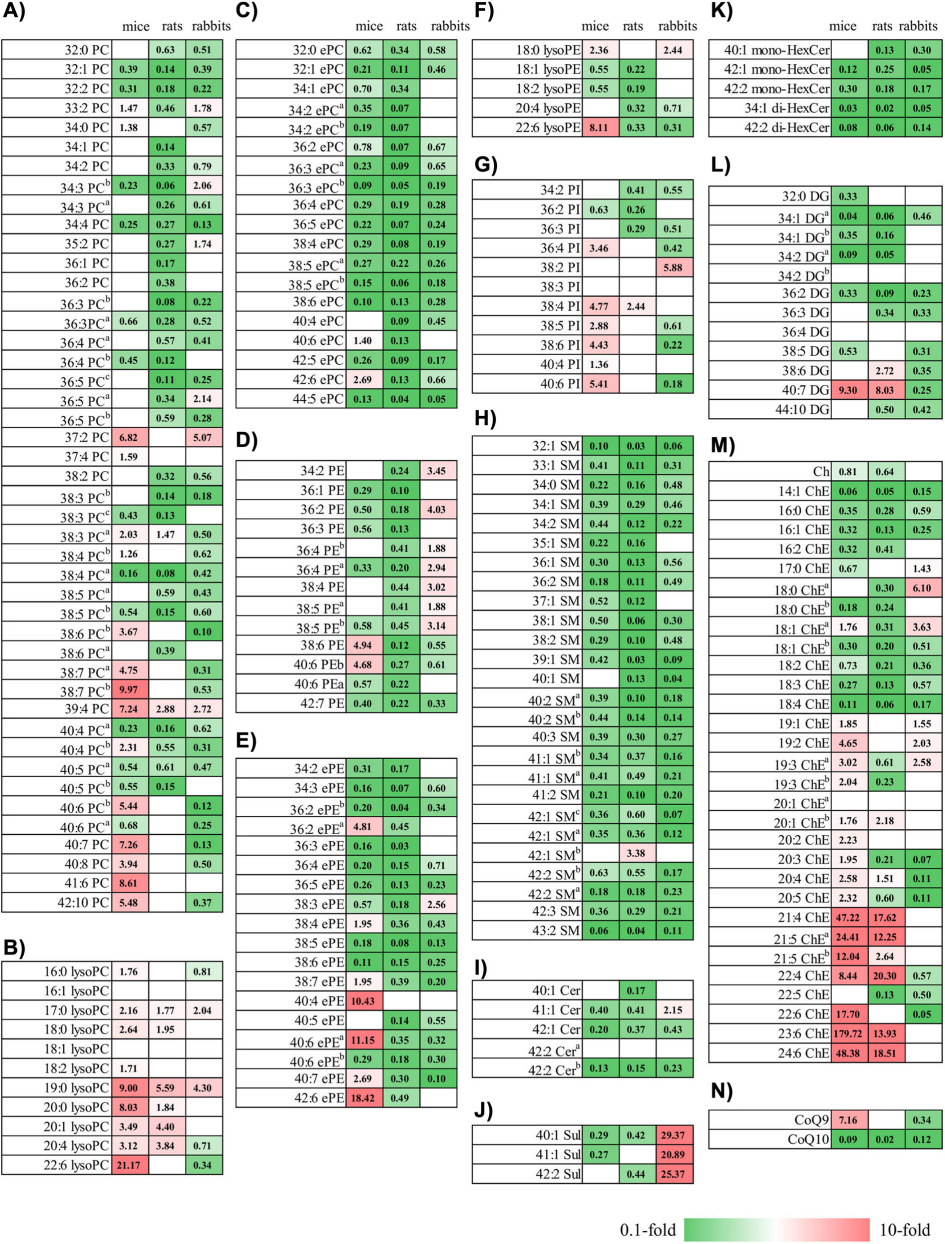
Heat maps of lipid metabolites among humans and three animal species. The heat map with significantly different (*p* < 0.05) lipid metabolites were generated using mean fold changes in the levels of metabolites calculated as ratios of each animal species to human. Vacant white cells indicate metabolites that were detected, but their levels were not significantly different. (**a**) PC, (**b**) lysoPC, (**c**) ePC, (**d**) PE, (**e**) ePE, (**f**) lysoPE, (**g**) PI, (**h**) SM, (**i**) Cer, (**k**) HexCer, (**l**) DG, (**m**) Ch/ChE and (**n**) CoQ. If more than two lipid species possessed the same chemical formula, alphabetical letters were put after each lipid molecule class to distinguish them from each other (Additional file 1: Table S1). PC, phosphatidylcholine; lysoPC, lysophosphatidylcholine; ePC, ether-type phosphatidylcholine; PE, phosphatidylethanolamine; lysoPE, lysophosphatidylethanolamine; ePE; ether-type phosphatidylethanolamine; PI, phosphatidylinositol; SM, sphingomyelin; Cer, ceramide; HexCer, hexosylceramide; DG, diacylglycerol; Ch/ChE, cholesterol/cholesteryl ester; CoQ, coenzyme Q

To investigate differences in circulating lipid profiles among humans and three animals often used in preclinical studies, we performed a non-targeted lipidomic analysis using plasma samples obtained from each mammal. To maintain experimental quality throughout extraction and measurement, the coefficient of variation for the IS was calculated as 11.7 %. In total, we identified 206 lipid metabolites consisting of 122 PLs, 39 SLs, and 45 NLs (Table 1 and Additional file 1: Table S1). Of the 206 identified lipid metabolites in all the mammals, the levels of 163, 166, and 151 metabolites were significantly different in mice, rats, and rabbits, respectively, when compared with the levels of those metabolites in humans (Fig. 1).

**Table 1:** Circulating lipid metabolites identified by non-targeted lipidomic analysis of each mammal

Lipid types	Ion mode	Lipid classes	Number
Phosphoglycerolipids	Negative	lysoPC	11
		lysoPE	5
		PC	45
		ePC	19
		PE	13
		ePE	18
		PI	11
Sphingolipids	Negative	SM	26
		Cer	5
		HexCer	5
		Sul	3
Neutral lipids	Positive	DG	12
		Ch/ChE	31
		CoQ	2
		Total	206

*lysoPC* lysophosphatidylcholine, *lysoPE* lysophosphatidylethanolamine, *PC* phosphatidylcholine, *ePC* ether-type phosphatidylcholine, *PE* phosphatidylethanolamine, *ePE* ether-type phosphatidylethanolamine, *PI* phosphatidylinositol, *SM* sphingomyelin, *Cer* ceramide, *HexCer* hexosylceramide, *DG* diacylglycerol, *Ch/ChE* cholesterol/cholesteryl ester, *CoQ* coenzyme Q

As shown in Fig. 1a, the levels of many phosphatidylcholines (PCs) were significantly lower in the three animals than in humans, although relatively longer and highly unsaturated PCs showed significantly higher levels in mice than in humans. The levels of many phosphatidylethanolamines (PEs) were significantly lower in rodents than in humans, while those of many PEs were significantly higher in rabbits than in humans (Fig. 1d). Among ether-type PCs and PEs (ePCs and ePEs), many metabolites showed significantly lower levels in the three selected animal models than in humans, with several exceptions in mice (Fig. 1c and e). Phosphatidylinositols (PIs) showed significantly higher levels in mice than in humans (Fig. 1g). In contrast, the levels of many PIs were significantly lower in rabbits than in humans. As shown in Fig. 1m, the levels of many ChEs were significantly different between humans and the selected animal species, although the differences varied in the three animal species.

Recently, lysoPCs and SLs have been proposed as biomarker candidates for the prediction and evaluation of diseased states and drug safety, respectively, such acetaminophen-induced liver injury [9], drug-induced phospholipidosis and β-cell dysfunction [12, 16] in preclinical studies. In the present study, most of the 11 lysoPCs (9 in mice and 6 in rats) were present at significantly higher levels in rodents than in humans (Fig. 1b). On the other hand, most of the 39 SLs (33 in mice, 36 in rats and 30 in rabbits) were present at significantly lower levels in all the three animal species which are often used in preclinical studies than in humans, although Suls showed significant higher levels in rabbits than in other mammals (Fig. 1h-k). These results indicated that broad molecules of lysoPCs and SLs show different levels in human and animal species. Therefore, species differences should be carefully considered when these lipid molecules are selected as biomarker candidates identified in preclinical studies for extrapolation and usage in clinical studies.

### Lipid metabolites showing more than 10-fold difference in their levels between humans and animal species

The extent of differences in the levels of lipid metabolites between humans and animal species that are used in preclinical studies seems to be the key problem for the extrapolation of biomarker candidates identified in preclinical studies into clinical studies. Because the range of fold change for the reported lipid biomarkers identified using animals species were approximately 0.5–8.0-fold [9, 12, 13, 16], the lipid metabolites that exceeded this range might be unsuitable as biomarker candidates in humans. We therefore focused on the lipid metabolites that showed greater than 10-fold value and significantly different levels in animal species as compared to their levels in humans (Tables 2, 3, 4). The numbers of these lipid metabolites were 19, 37, and 12 for mice, rats, and rabbits, respectively.

**Table 2:** Lipid metabolites that showed more than 10-fold and significant differences between humans and mice

>10-fold		<10-fold	
Metabolite	Fold	Metabolite	Fold
22:6 lysoPC	21.1692	36:3 ePC^b^	0.0887
21:4 ChE	47.2233	38:6 ePC	0.0973
21:5 ChE^a^	24.4064	43:2 SM	0.0648
21:5 ChE^b^	12.0447	34:1 di-HexCer	0.0293
22:6 ChE	17.7039	42:2 di-HexCer	0.0790
23:6 ChE	179.7175	34:1 DG^a^	0.0409
24:6 ChE	48.3842	34:2 DG^a^	0.0935
40:4 ePE	10.4272	14:1 ChE	0.0610
40:6 ePE^a^	11.1450	CoQ10	0.0899
42:6 ePE	18.4164		

If more than two lipid species possessed the same chemical formula, alphabetical letters were put after each lipid molecule class to distinguish them from each other (Additional file 1: Table S1)

*lysoPC* lysophosphatidylcholine, *ChE* cholesteryl ester, *ePE* ether-type phosphatidylethanolamine, *ePC* ether-type phosphatidylcholine, *SM* sphingomyelin, *HexCer* hexosylceramide, *DG* diacylglycerol, *CoQ* coenzyme Q

**Table 3:** Lipid metabolites that showed more than 10-fold and significant differences between humans and rats

>10-fold		<10-fold	
Metabolite	Fold	Metabolite	Fold
21:4 ChE	17.6192	34:3 PC^b^	0.0648
21:5 ChE^a^	12.2477	36:3 PC^b^	0.0827
22:4 ChE	20.3018	38:4 PC^a^	0.0844
23:6 ChE	13.9319	34:2 ePC^a^	0.0717
24:6 ChE	18.5058	34:2 ePC^b^	0.0713
		36:2 ePC	0.0724
		36:3 ePC^a^	0.0934
		36:3 ePC^b^	0.0516
		36:5 ePC	0.0729
		38:4 ePC	0.0782
		38:5 ePC^b^	0.0600
		40:4 ePC	0.0877
		42:5 ePC	0.0947
		44:5 ePC	0.0411
		36:1 PE	0.0997
		34:3 ePE	0.0656
		36:2 ePE^b^	0.0409
		36:3 ePE	0.0331
		38:5 ePE	0.0778
		32:1 SM	0.0293
		38:1 SM	0.0626
		39:1 SM	0.0339
		40:2 SM^a^	0.0957
		43:2 SM	0.0401
		34:1 di-HexCer	0.0186
		42:2 di-HexCer	0.0643
		34:1 DG^a^	0.0648
		34:2 DG^a^	0.0523
		36:2 DG	0.0945
		14:1 ChE	0.0513
		18:4 ChE	0.0565
		CoQ10	0.0174

If more than two lipid species possessed the same chemical formula, alphabetical letters were put after each lipid molecule class to distinguish them from each other (Additional file 1: Table S1)

*ChE* cholesteryl ester, *PC* phosphatidylcholine, *ePC* ether-type phosphatidylcholine, *PE* phosphatidylethanolamine, *ePE* ether-type phosphatidylethanolamine, *SM* sphingomyelin, *HexCer* hexosylceramide, *DG* diacylglycerol, *CoQ* coenzyme Q

**Table 4:** Lipid metabolites that showed more than 10-fold and significant differences between humans and rabbits

>10-fold		<10-fold	
Metabolite	Fold	Metabolite	Fold
40:1 Sul	29.3688	44:5 ePC	0.0541
41:1 Sul	20.8891	32:1 SM	0.0551
42:2 Sul	25.3672	39:1 SM	0.0858
		40:1 SM	0.0402
		42:1 SM^c^	0.0701
		42:1 mono-HexCer	0.0465
		34:1 di-HeXCer	0.0475
		20:3 ChE	0.0666
		22:6 ChE	0.0528

If more than two lipid species possessed the same chemical formula, alphabetical letters were put after each lipid molecule class to distinguish them from each other (Additional file 1: Table S1)

*Sul* sulfatide, *ePC* ether-type PC, *SM* sphingomyelin, *HexCer*, hexosylceramide, *ChE* cholesteryl ester

Interestingly ChEs with PUFA, such as 23:6 and 24:6 ChE, showed markedly higher levels (179.7175- and 48.3842-fold, respectively) in mice than in humans (Table 2). These large changes of the PUFA-containing ChEs were also consistent with the results obtained in rats, showing 13.9319-fold and 18.5058-fold changes for 23:6 and 24:6 ChE, respectively (Table 3). These results indicated that the levels of circulating PUFA-containing ChEs are higher in rodents than in humans. However, the mechanism underlying the species-specific difference in these levels remains unknown.

As shown in Table 3, 15 ether-type PLs, such as 36:3 ePC^b^ and 36:3 ePE, were present in drastically lower levels (less than 0.1-fold) in rats as compared to their levels in humans (the range of fold changes; 0.0331-0.0947). In addition, all other ether-type PLs, except 40:4 ePE, were significantly lower in rats than in humans (Fig. 1c and e). Moreover, many ether-type PLs showed significantly lower levels in mice than in humans (Fig. 1c and e), although the ether-type PLs which showed less than 0.1-fold change and significantly lower levels were only found for 36:3 ePC^b^ and 38:6 ePC (Table 2). These observations suggested that circulating basal levels of many ether-type PLs are lower in rodents than in humans. The mechanism underlying this difference in the levels of ether-type PLs in rodents remains unknown and needs to be investigated in the future studies.

Rabbits are often used in preclinical studies in addition to rodents. All three Suls showed more than 20-fold higher levels in rabbits than in humans (Table 4). Suls have been proposed as biomarkers for neurodegenerative diseases such as Alzheimer’s disease [17] and multiple sclerosis [18]. Therefore, when Suls are identified as biomarker candidates for disease states and drug responses in rabbits, their extrapolation into human clinical trials calls for extensive study and needs attention.

## Conclusions

We compared the profiles of circulating lipid metabolites between humans and three animal species often used in preclinical studies by using a non-targeted lipidomics approach. By comparing these profiles, we revealed that some of circulating lipid metabolites were present at markedly different levels between humans and animal species. Our results therefore provide useful and fundamental information for selecting biomarker candidates that might be identified in future preclinical studies for their successful extrapolation into human clinical studies.

## Additional file

Additional file 1: Table S1. Normalized peak height of phosphoglycerolipids, sphingolipids, and neutral lipids in the plasma of all the mammals. (XLSX 112 kb)

## Abbreviations

PLs: Phosphoglycerolipids
SLs: Sphingolipids
lysoPC: Lysophosphatidylcholine
LC-MS: Liquid chromatography-mass spectrometry
PUFA: Poly-unsaturated fatty acid
ChE: Cholesteryl ester
Sul: Sulfatide
PE: Phosphatidylethanolamine
IS: Internal standard
NLs: Neutral lipids
TOF: Time-of flight
PC: Phosphatidylcholine
PI: Phosphatidylinositol
ePC: Ether-type phosphatidylcholine
ePE: Ether-type phosphatidylethanolamine
